# Paracrine interactions through FGFR1 and FGFR2 receptors regulate the development of preimplantation mouse chimaeric embryo

**DOI:** 10.1098/rsob.220193

**Published:** 2022-11-16

**Authors:** Katarzyna Krawczyk, Katarzyna Wilczak, Katarzyna Szczepańska, Marek Maleszewski, Aneta Suwińska

**Affiliations:** Department of Embryology, Institute of Developmental Biology and Biomedical Sciences, Faculty of Biology, University of Warsaw, Miecznikowa 1, 02-096 Warsaw, Poland

**Keywords:** early mammalian development, FGF4/ERK signalling pathway, cell fate specification, developmental plasticity, chimaeric embryo

## Abstract

The preimplantation mammalian embryo has the potential to self-organize, allowing the formation of a correctly patterned embryo despite experimental perturbation. To better understand the mechanisms controlling the developmental plasticity of the early mouse embryo, we used chimaeras composed of an embryonic day (E)3.5 or E4.5 inner cell mass (ICM) and cleaving 8-cell embryo. We revealed that the restricted potential of the ICM can be compensated for by uncommitted 8-cell embryo-derived blastomeres, thus leading to the formation of a normal chimaeric blastocyst that can undergo full development. However, whether such chimaeras maintain developmental competence depends on the presence or specific orientation of the polarized primitive endoderm layer in the ICM component. We also demonstrated that downregulated FGFR1 and FGFR2 expression in 8-cell embryos disturbs intercellular interactions between both components and results in an inverse proportion of primitive endoderm and epiblast within the resulting ICM and abnormal embryo development. This finding suggests that FGF signalling is a key part of the regulatory mechanism that assigns cells to a given lineage and ensures the proper composition of the blastocyst, which is a prerequisite for its successful implantation in the uterus and for further development.

## Graphical abstract

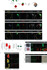

## 1. Introduction

During mammalian preimplantation development, cells of the embryo ‘make decisions’ concerning their fate, resulting in the gradual loss of their totipotency (i.e. the ability to give rise to the trophectoderm (TE), primitive endoderm (PE) and epiblast (EPI)), and they undergo specialization. The blastocyst's inner cell mass (ICM) cells lose their plasticity successively, first failing to form the TE and then restricting their fate to either the PE or the EPI within the ICM [1–7]. However, despite the reduced cell potency, the embryo as a whole retains a high degree of developmental plasticity, which guarantees the proper course of development even in the face of severe perturbations [8,9]. These regulatory abilities of early mammalian embryos have laid the groundwork for the creation of chimaeric animals and for preimplantation genetic diagnosis in humans, among other applications; however, the mechanisms underlying this developmental plasticity remain unclear.

Since the FGF4/ERK (*fibroblast growth factor/extracellular signal-regulated kinase*) pathway has been shown to play an important role in balance between PE and EPI cell fate specification within the ICM [4,10–12] and the restriction of ICM potency [7], we aimed to assess whether this signalling underlies the regulatory nature of mammalian embryos. Several FGF ligands are expressed in the preimplantation mouse embryo, but *Fgf4* is the first gene to have bimodal expression within the ICM at embryonic day (E)3.25 [13,14]. It was demonstrated that the binding of FGF4 to its receptors, FGFR1 and FGFR2, activates the ERK pathway to induce the PE fate by maintaining GATA6 and concomitantly downregulating NANOG protein expression [10,11,15–18]. It has also been shown that genetic ablation of FGF signalling [10,11,16,18,19], as well as pharmacological inhibition of FGF activity [20,21], directs ICM cells towards an EPI fate. Conversely, treating embryos with exogenous FGF4 prevents the development of the EPI and results in all ICM cells adopting a PE identity [21].

The expression of FGFR1 and FGFR2 has also been detected in TE cells [18,22,23]. Thus, it was proposed that paracrine interactions through FGF receptors affect the development of both the PE and TE extraembryonic lineages. Together, these findings call for a reassessment of how the FGF4/ERK pathway can regulate the developmental plasticity of the mouse preimplantation embryo.

To gain deeper insights into the mechanism controlling the plasticity of the early mouse embryo, we used a chimaeric embryo as a model to disrupt the intercellular interactions between its components. Bearing in mind that the FGF4 signal is produced by EPI cells and that the cells of the cleaving embryo have full developmental potential and are also sensitive to FGF4 stimulation [21,24], we constructed chimaeric embryos composed of the ICM of the E3.5 or E4.5 blastocyst and 8-cell embryo and tracked the development of the aggregates before implantation and following their transfer into the recipient mouse. We showed that until the late blastocyst stage, the ICM can integrate with the 8-cell embryo and that as long as these components are maintained in a strictly defined configuration, they can reconstruct a normal blastocyst that is able to undergo full development. We also demonstrated that ICM cells, through the FGF4 signal, direct the progeny of the 8-cell embryo to preferentially contribute to the TE and PE extraembryonic lineages. Additionally, we found that the simultaneous downregulation of *Fgfr1* and *Fgfr2* in the 8-cell mouse embryo disturbs intercellular interactions between its cells and the E3.5 ICM, leading to an inverse proportion of PE and EPI cells within the ICM of the resulting chimaeric embryo, and thus abnormal embryo development. This finding suggests that the FGF4/ERK signalling pathway is required to ensure the proper formation of all three cell lineages of the blastocyst (the EPI, PE and TE) and constitutes a part of the regulatory mechanism underlying the plasticity of the preimplantation mouse embryo.

## 2. Results

To study the developmental potency of the ICM, we used chimaeric embryos (i.e. embryos composed of genetically distinct cells derived from two zygotes). As it was evidenced that ICMs already have limited plasticity [3,7], we aggregated them with 8-cell embryos, whose cells have full potential to differentiate into all cell lineages of the blastocyst.

### 2.1. Preimplantation development of a chimaeric embryo obtained by aggregating an 8-cell embryo with an E3.5 inner cell mass

First, we examined whether the E3.5 ICM is capable of integrating with an 8-cell embryo and co-creating a normal chimaeric embryo. To distinguish the progeny of both components, we used 8-cell embryos expressing red fluorescence protein (RFP) and ICMs expressing green fluorescence protein (GFP). The aggregates were cultured until the blastocyst stage and then analysed using specific antibodies against the markers of the PE (GATA4) and TE (CDX2) lineages. Control E3.5 blastocysts, which were not subjected to any manipulations, were used to determine the stage of development of the experimental blastocysts from which the ICM was isolated. They were fixed at the same time as when the ICMs were isolated. The total number of cells in the control blastocysts (*n* = 24) was 59.5 ± 4.7, whereas the number of TE and ICM cells averaged 34.6 ± 3.3 and 24.9 ± 5.5, respectively (electronic supplementary material, table S1). In line with earlier observations [4,14,19,25–28], the blastocysts at this stage of development contained EPI and PE progenitors exhibiting mutually exclusive expression of SOX2 and SOX17, respectively, alongside cells coexpressing both of these markers (electronic supplementary material, figure S1).

**Figure 1.**
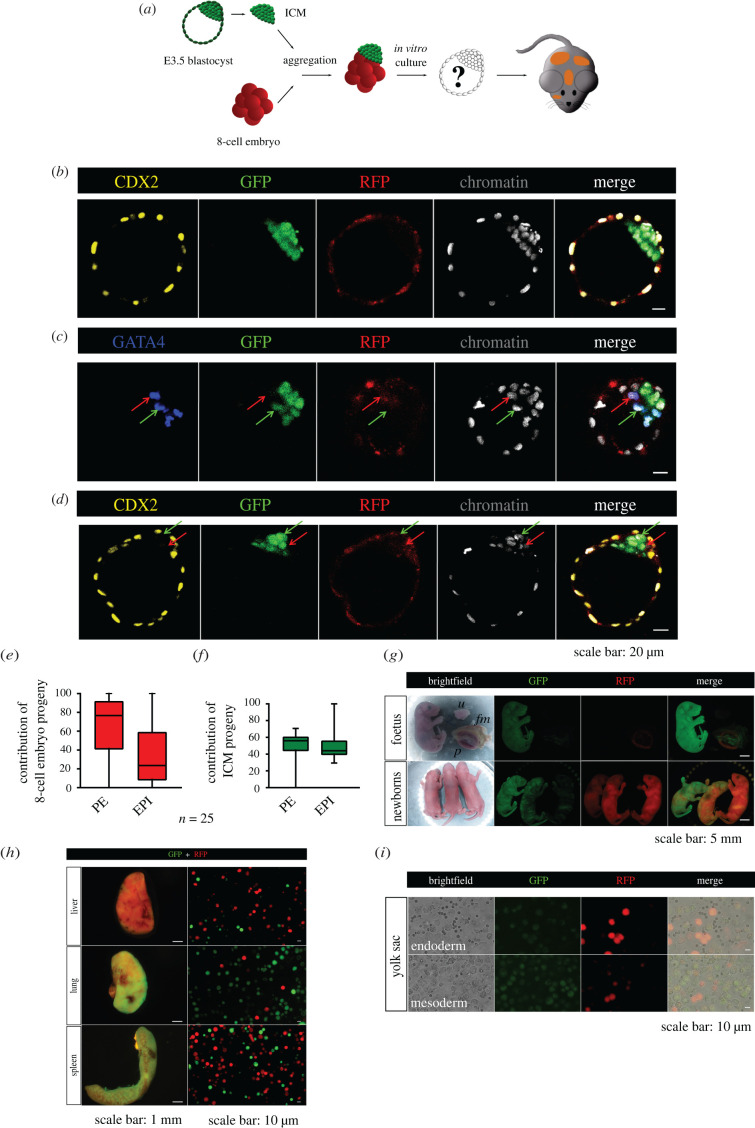
Development of chimaeric embryos obtained by aggregation of an 8-cell embryo with an E3.5 ICM. (*a*) Schematic representation of the experimental strategy used. (*b–d*) The detection of CDX2 (yellow), GATA4 (blue), GFP (green) and RFP (red) in representative chimaeric blastocysts. (*b*) A blastocyst with the TE containing cells derived only from the 8-cell embryo. (*c*,*d*) The blastocysts in which the PE and EPI originated from the progeny cells of both components. The red arrows indicate cells derived from the 8-cell embryo, while the green arrows indicate the ICM progeny. (*e*,*f*) The contributions of the progeny of the 8-cell embryo and ICM to the PE and EPI lineages. The graphs present medians and the first and third quartile values. The ends of the whiskers show the minimum and maximum values. (*g*) E19.0 fetus and newborns displaying chimaerism evidenced by the presence of GFP- and RFP-expressing cells in tissues (*p*—placenta, *fm*—fetal membranes, *u*—control mother's uterus). (*h*) Chimaerism in the liver, lung and spleen. (*i*) Cell suspension after disaggregation of the endodermal and mesodermal layers of the yolk sac, confirming its chimaerism (erythrocytes do not show fluorescence).

ICMs isolated from the experimental blastocysts were aggregated with 8-cell embryos (figure 1*a*). We obtained 25 aggregates (in four experiments), of which all developed *in vitro* to the blastocyst stage within 48 h. Time-lapse recording allowed us to follow their development from the moment of aggregation until they reached the blastocyst stage (electronic supplementary material, movie S1). We found that within a few hours after aggregation, the progeny cells of both components were gradually integrating. We observed that ICM cells localized inside the aggregate and then the entire embryo compacted. After 24 h of culture, we noted the first signs of cavitation, and after the next 24 h, the embryos reached the late blastocyst stage. These chimaeric blastocysts had an average of 146.3 ± 14.9 cells, with 108.8 ± 14.7 cells in the TE and 37.5 ± 5.6 cells in the ICM. The chimaeric blastocysts' ICMs contained an average of 21.2 ± 4.5 PE cells and 16.3 ± 4.4 EPI cells (electronic supplementary material, table S2).

Next, we checked the localization of the progeny of the 8-cell embryo and ICM cells of the E3.5 blastocysts in the resulting chimaeric embryos. In all embryos, the TE was composed exclusively of the progeny of the 8-cell stage blastomeres (figure 1*b*). In turn, the PE layer most often consisted of both ICM and 8-cell embryo progeny (18 of 25 blastocysts; 72%; figure 1*c*; electronic supplementary material, figure S2). Less commonly, we observed chimaeric blastocysts in which the PE contained either cells derived only from the ICM (6 of 25 blastocysts; 24%) or cells from only the 8-cell embryo (1 of 25 blastocysts; 4%). EPI cells in the chimaeric embryos originated mostly from the progeny cells of both components (18 of 25 blastocysts; 72%; figure 1*d*; electronic supplementary material, figure S2). Less frequently observed were blastocysts with the EPI consisting solely of ICM-derived cells (7 of 25 blastocysts; 28%).

Among the ICM cells that originated from the progeny of the 8-cell embryo, 62.3% ± 35.9% created the PE, while 37.7% ± 35.9% contributed to the EPI lineage (figure 1*e*). By contrast, the progeny of the E3.5 ICM formed both ICM lineages equally in the chimaeric blastocysts: 49.7% ± 17.6% of PE cells and 50.3% ± 17.6% of EPI cells (figure 1*f*).

Taken together, our results indicate that the E3.5 ICM lost the ability to differentiate into the TE and formed only the EPI and PE in the chimaeric embryo. Importantly, the ICM-restricted potency did not affect the plasticity of the entire chimaeric embryo, since following the aggregation with the 8-cell embryo and further development, both components were able to form a morphologically normal chimaeric blastocyst. However, the presence of the ICM restricted the fate choice of the progeny of the 8-cell embryo, as evidenced by its preferential contribution to the TE and PE lineages.

### 2.2. Postimplantation development of a chimaeric embryo obtained by aggregating an 8-cell embryo with an E3.5 inner cell mass

Since we found that the aggregates composed of the 8-cell embryo and E3.5 ICM could develop properly to the blastocyst stage, we next investigated whether they would be able to undergo full embryogenesis until birth. To this aim, we transferred 34 chimaeric embryos (obtained in five experiments) into the oviducts of female recipients (*n* = 6). The transfers resulted in 10 fetuses/newborns and 1 resorption. The efficiency of this procedure, which we defined as the quotient of the number of implanted embryos to the number of transplanted embryos, was 32.3%. The obtained fetuses and newborns differed in the distribution of RFP- and GFP-expressing cells (figure 1*g*). To determine the contribution of the progeny of both components of the chimaeric embryos to the formation of embryonic and extraembryonic tissues, we disaggregated selected organs and examined the presence of fluorescence. We found that all tissues were composed of both progeny of the 8-cell embryo and the ICM of the E3.5 blastocyst (figure 1*h*). We also noticed that the yolk sac consisted of both components of the chimaeric embryo (figure 1*i*), while the placenta consisted exclusively of the 8-cell embryo progeny.

In summary, these results showed that after transfer to pseudopregnant recipients, the aggregates composed of the 8-cell embryo and E3.5 ICM could develop to term and produce healthy chimaeric offspring. The progeny of the ICM did not contribute to the placenta (TE derivative), but was preferentially located in the internal organs and also in the mesoderm and endoderm of the yolk sac (EPI and PE derivatives, respectively).

### 2.3. Preimplantation development of a chimaeric embryo obtained by aggregating an 8-cell embryo with an E4.5 inner cell mass

Next, we investigated whether E4.5 ICMs are capable of reconstructing normal chimaeric blastocysts following aggregation with the 8-cell embryo. Control E4.5 blastocysts had an average of 109.4 ± 12.5 cells, whereas the mean numbers of TE and ICM cells were 78.6 ± 8.3 and 30.8 ± 7.3, respectively (electronic supplementary material, table S3). The ICMs of such blastocysts contained on average 18.4 ± 4.9 PE cells and 12.4 ± 3.3 EPI cells, which were restricted to their respective layers (electronic supplementary material, table S3 and figure S3).

**Figure 2.**
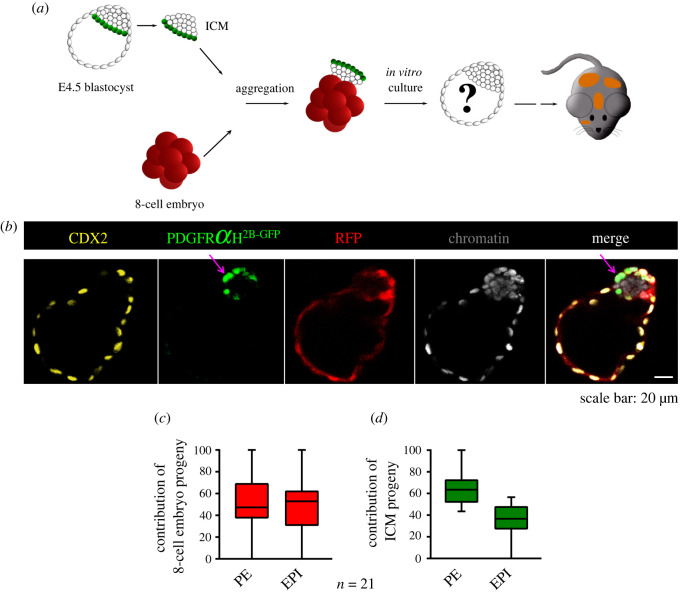
Development of aggregates composed of an 8-cell embryo in contact with the EPI of the E4.5 ICM. (*a*) Experimental outline of chimaera aggregation assay. (*b*) CDX2 (yellow), PDGFR*α*^H2B-GFP^ (green) and RFP (red) localization in a representative abnormal chimaeric blastocyst. The purple arrow indicates the layer of the PE that partially contributes to the TE. (*c,d*) The contributions of the progeny of the 8-cell embryo and ICM to the PE and EPI lineages. The graphs present medians and the first and third quartile values. The ends of the whiskers show the minimum and maximum values.

**Figure 3.**
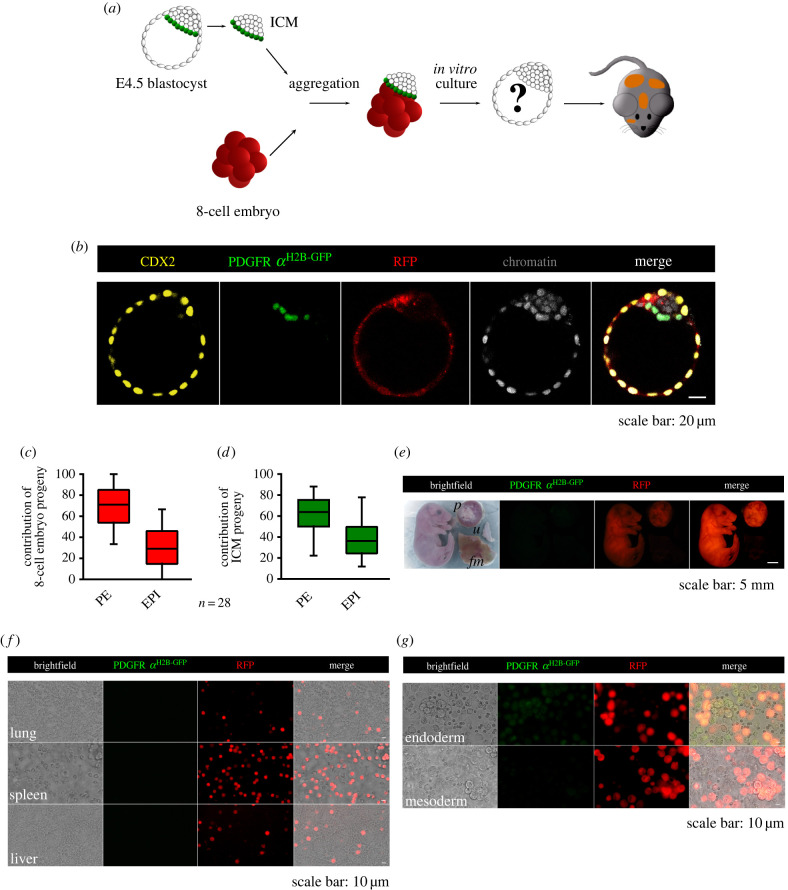
Development of aggregates composed of an 8-cell embryo in contact with the PE of the E4.5 ICM. (*a*) Scheme illustrating the experimental protocol used. (*b*) Representative confocal images of a chimaeric blastocyst showing the localization of cells expressing CDX2 (yellow), PDGFR*α*^H2B-GFP^ (green) and RFP (red). (*c*,*d*) The contributions of the progeny of the 8-cell embryo and ICM to the PE and EPI lineages. The graphs present medians and the first and third quartile values. The ends of the whiskers show the minimum and maximum values. (*e*) Chimaeric neonate with the placenta (*p*), fetal membranes (*fm*) and control mother's uterus (*u*). (*f*,*g*) Cell suspensions of the lung, spleen, liver and yolk sac.

Since the E4.5 ICM cells were already segregated and spatially separated, we hypothesized that the successful development of chimaeras might depend on the orientation of the ICM in an aggregate in relation to the 8-cell embryo. To test this hypothesis, we constructed two types of aggregates in which either EPI or PE cells were in direct contact with the RFP-expressing 8-cell embryo (figures 2*a*, 3*a*). This construction was possible since E4.5 blastocysts—the donors of the ICMs—express GFP protein under the control of the *Pdgrfα* gene promoter, which is active only in PE cells.

#### 2.3.1. Preimplantation development of aggregates composed of an 8-cell embryo in contact with the epiblast of an E4.5 inner cell mass

We found that all chimaeric aggregates (*n* = 21; obtained in four experiments) in which the 8-cell embryos were in contact with the EPI cells of the E4.5 ICMs developed into abnormal chimaeric blastocysts, with the PE layer placed outside the embryo at the site of the TE (figure 2*b*; electronic supplementary material, movie S2, figure S4). We noted that the inappropriately located cells of the PE lineage were formed by ICM progeny. The mean total number of cells in such chimaeric blastocysts was 148.1 ± 6.1 cells, while the mean numbers of TE and ICM cells were 99.4 ± 5.1 and 48.7 ± 8.9, respectively. The ICM contained on average 32.8 ± 7.5 PE cells (including those atypically positioned on the outside of the embryo) and 15.9 ± 6.5 EPI cells (electronic supplementary material, table S4).

We noticed that in all chimaeric blastocysts, the TE consisted entirely of 8-cell embryo-derived cells. The most common were blastocysts whose PE was composed of both the 8-cell embryo progeny and PE cells derived from the E4.5 ICM (18 of 21 blastocysts, 85.7%; electronic supplementary material, figure S4). Less frequently we observed blastocysts in which the entire PE lineage was derived only from this lineage of E4.5 ICMs (3 of 21 blastocysts; 14.3%). Similarly, the EPI was most often formed by cells derived from both components of a chimaeric embryo (the 8-cell embryo and the EPI of the E4.5 ICM; 18 of 21 blastocysts, 85.7%). We seldom noted blastocysts with the EPI composed solely of EPI cells of the E4.5 ICM (3 of 21 blastocysts; 14.3%).

Then, we examined the contributions of the progeny of both components of the ICM lineages. The progeny of the 8-cell embryo contributed equally to the PE and EPI lineages, averaging 53% ± 24.2% and 47% ± 24.2%, respectively (figure 2*c*). By contrast, the average proportions of E4.5 ICM progeny constituting the PE and EPI were 64.4% ± 13.9% and 35.6% ± 13.9%, respectively (figure 2*d*).

#### 2.3.2. Postimplantation development of aggregates composed of an 8-cell embryo in contact with the epiblast of an E4.5 inner cell mass

To investigate whether the aggregate composed of the E4.5 ICM with the EPI directed to an 8-cell embryo could continue its development within the uterus, we transferred 57 chimaeric embryos (obtained in 10 experiments) to the oviducts of female recipients (*n* = 8). We found that these aggregates were unable to undergo full development. In the uteruses isolated from pseudopregnant females, we found 16 implantation sites with fetal remains (transplantation efficiency: 28.1%). This result suggests that in contrast with E3.5 ICMs, ICMs isolated from E4.5 blastocysts have limited developmental potency in aggregates in which EPI cells were in direct contact with the 8-cell embryo.

#### 2.3.3. Preimplantation development of aggregates composed of an 8-cell embryo in contact with the primitive endoderm of an E4.5 inner cell mass

To examine whether the position of the components in the aggregates influences their developmental potential, we created aggregates in which the PE of the ICM was adjacent to an 8-cell embryo. Of the 30 constructed aggregates (obtained in five experiments), almost all (28 of 30; 93.3%) developed into morphologically normal chimaeric blastocysts (figure 3*b*; electronic supplementary material, movie S3). In the remaining two cases, the blastocysts showed irregularities in the formation of cell lineages (not shown).

For further analysis, we used only the chimaeric blastocysts with undisturbed morphology. The mean cell number of these chimaeras was 176 ± 17.2 cells, of which 115 ± 13.1 cells constituted the TE, while the remaining cells formed the ICM (61 ± 11.1 cells), which consisted of 41.1 ± 9.2 PE and 19.9 ± 6.9 EPI cells (electronic supplementary material, table S5).

We noted that the TE of all analysed embryos (*n* = 28) was composed of 8-cell embryo-derived cells. In turn, the PE and EPI of all chimaeric blastocysts consisted of both the 8-cell embryo progeny and ICM cells derived from E4.5 blastocysts. We observed that 8-cell embryo progeny more readily contributed to the PE than the EPI lineage (70.3% ± 19.8% and 29.7% ± 19.8%, respectively; figure 3*c*). Similarly, the descendants of the E4.5 ICM were preferentially located in the PE and averaged 60.5% ± 17.7%, while in the EPI lineage they represented only 39.5% ± 17.7% of the total ICM (figure 3*d*).

#### 2.3.4. Postimplantation development of aggregates composed of an 8-cell embryo in contact with the primitive endoderm of an E4.5 inner cell mass

Our next step was to explore whether the aggregates composed of the E4.5 ICM with the PE directed to an 8-cell embryo could undergo whole embryogenesis after transplantation into the recipient mouse. To this aim, we constructed 54 chimaeric aggregates (obtained in nine experiments) and subsequently performed transfers (*n* = 8) that resulted in 16 fetuses (figure 3*e*) and 1 resorption (transplantation efficiency: 31.5%). Then, we isolated embryonic and extraembryonic tissues from all obtained fetuses and found that the organs were built by the progeny of the 8-cell embryo and EPI cells derived from the ICM of the E4.5 blastocyst (figure 3*f*). The endoderm layer of the yolk sac originated from progeny of both the 8-cell embryo and PE cells, while the mesoderm layer was composed of 8-cell embryo-derived cells and EPI cells of the E4.5 ICM (figure 3*g*). By contrast, the placenta consisted entirely of descendants of the 8-cell embryo (figure 3*e*).

To sum up, our results show that the ICM of the E4.5 blastocyst, similarly to the E3.5 ICM, exhibited restricted developmental plasticity since its progeny form only EPI and PE lineages. By constructing two types of aggregates with different orientations of the PE and EPI layers to the 8-cell embryo, we showed that the successful development of the chimaeric embryo is possible only when the 8-cell embryo is positioned in direct contact with the PE layer.

### 2.4. Preimplantation development of chimaeric embryos obtained by aggregating an 8-cell embryo with an E4.5 inner cell mass consisting solely of epiblast cells

**Figure 4.**
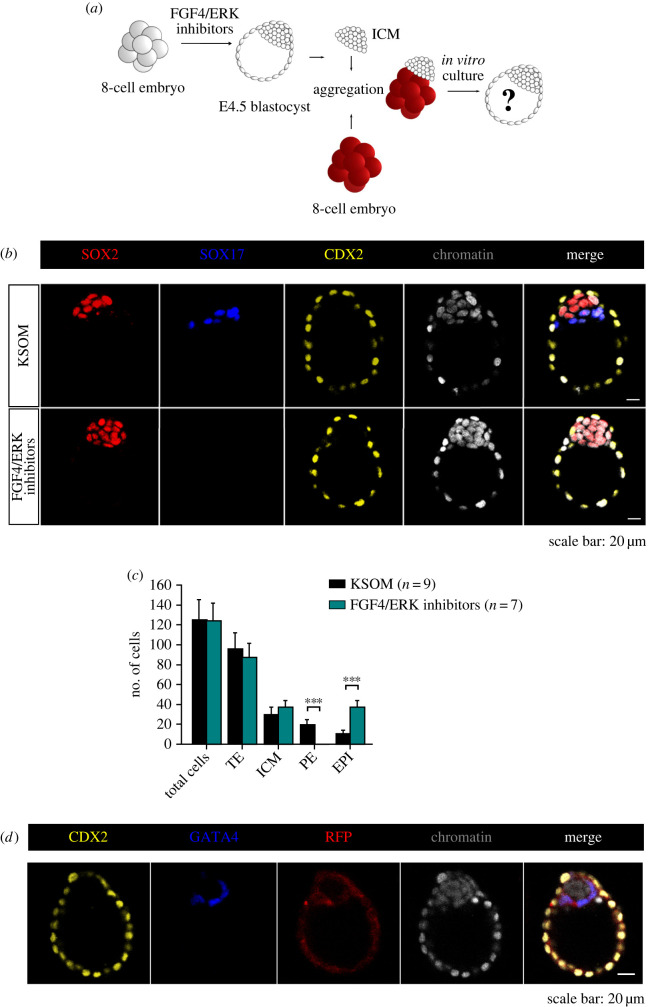
Development of chimaeric embryos obtained by aggregation of an 8-cell embryo with an E4.5 ICM consisting solely of EPI cells. (*a*) Schematic of the time schedule of FGF4/ERK inhibitors treatment and chimaera formation. (*b*) The expression of SOX2 (red), SOX17 (blue) and CDX2 (yellow) in representative blastocysts developed from embryos cultured in KSOM medium and embryos treated with FGF4/ERK inhibitors. (*c*) Cell numbers of blastocyst lineages in embryos cultured in inhibitor-free medium and embryos cultured in medium with FGF4/ERK inhibitors. ***p < 0.001. Data are represented as mean ± s.d. (*d*) Detection of CDX2 (yellow), GATA4 (blue) and RFP (red) in the representative chimaeric blastocyst.

The results of our experiments showed that the spatial arrangement of the components in the aggregates plays an important role in their further development. To check whether this developmental restriction is related to the polarization of the ICM, we aggregated the 8-cell embryo with an E4.5 ICM composed exclusively of EPI cells (figure 4*a*). To obtain blastocysts devoid of PE cells, we cultured the embryos from the 8-cell stage to the late E4.5 blastocyst stage in the presence of FGF4/ERK pathway inhibitors [20,21]. To ensure that the pharmacological inhibition of FGF4/ERK activity effectively blocked the formation of PE cells, we created two control groups. The first group consisted of embryos cultured in the medium without inhibitors (*n* = 9), while the second group consisted of embryos treated with inhibitors (*n* = 7; figure 4*b*). Blastocysts from both groups were composed of a similar number of cells: 124.1 ± 21.5 and 123.5 ± 18.3 cells, respectively (figure 4*c*). The average numbers of TE and ICM cells in the blastocysts obtained from unmanipulated embryos were 94.7 ± 17.5 and 29.4 ± 8.0, respectively, and these numbers were not significantly different from those of the corresponding lineages in the blastocysts that had developed from embryos treated with FGF4/ERK pathway inhibitors: 86.6 ± 14.9 and 36.9 ± 7.1, respectively (figure 4*c*). As expected, upon FGF inhibition, we observed that all ICM cells adopted an EPI fate (figure 4*b*). Conversely, untreated embryos contained on average 19.3 ± 5.2 PE cells (*p* < 0.0001; figure 4*b,c*). The mean numbers of EPI cells in the blastocysts that had developed from embryos cultured in inhibitor-free medium and in the blastocysts that had developed from embryos cultured in medium supplemented with inhibitors were 10.1 ± 4.2 and 36.9 ± 7.1, respectively (*p* < 0.0001; figure 4*c*).

Having tested the inhibitors of the FGF4/ERK pathway, we created 37 chimaeric aggregates (in six experiments) composed of an 8-cell embryo and an E4.5 ICM consisting solely of EPI cells. We found that all of such chimaeras developed into morphologically normal blastocysts with the PE being of 8-cell embryo origin (figure 4*d*). Chimaeric blastocysts had an average of 101 ± 18.7 cells, with 74.6 ± 17.3 cells in the TE and 26.4 ± 3.8 cells in the ICM. Their ICMs contained on average 16.4 ± 4.5 PE cells and 10 ± 4.4 EPI cells.

This finding suggests that the presence of polar PE cells outside the aggregates decreases the regulatory capabilities of chimaeric embryos composed of 8-cell embryos being in contact with the EPI of E4.5 ICMs.

### 2.5. Effect of inhibiting FGF4/ERK signalling on mouse embryo development and its plasticity

**Figure 5.**
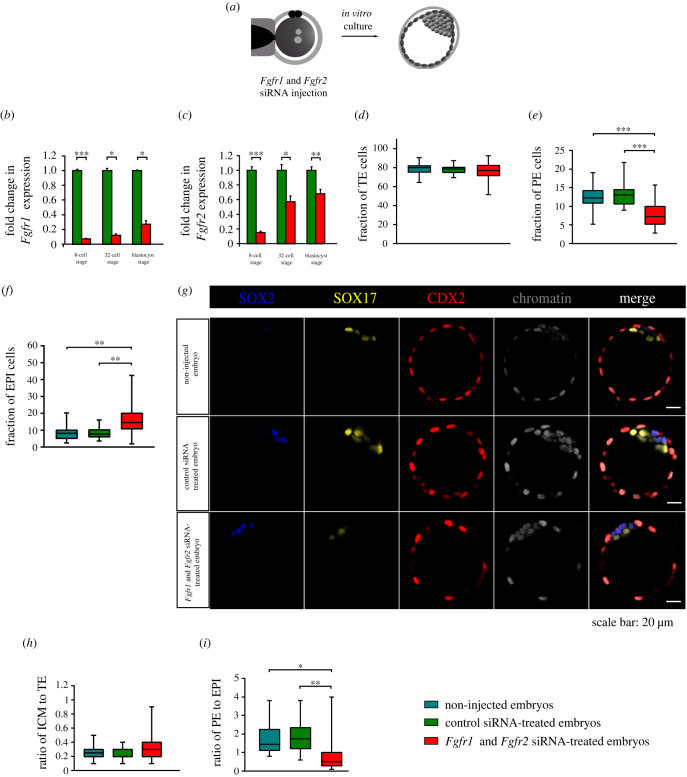
Effect of *Fgfr1* and *Fgfr2* knockdown on lineage specification at E4.5. (*a*) Scheme of *Fgfr1* and *Fgfr2* siRNA experiment. RT-PCR-derived relative *Fgfr1* (*b*) and *Fgfr2* (*c*) transcript levels between control siRNA-treated embryos and *Fgfr1* and *Fgfr2* siRNA-treated embryos at the 8-cell stage, the 32-cell stage and the E4.5 blastocyst stage. Error bars indicate s.d. (*d*–*f*) Quantitative analysis of the percentage of CDX2+ cells (TE), as well as the percentages of SOX17+ (PE) and SOX2+ (EPI) cells. (*g*) Immunodetection of SOX2 (blue), SOX17 (yellow) and CDX2 (red) in representative blastocysts. The ICM to TE ratio and the PE to EPI ratio are shown in (*h*) and (*i*), respectively. For (*d–f,h*,*i*), the graphs present medians and the first and third quartile values. The ends of the whiskers show the minimum and maximum values. *p < 0.05, **p < 0.01, ***p < 0.001.

We established that the chimaeric embryo composed of the 8-cell embryo and an ICM preserves its regulatory capabilities despite the difference in the developmental progress of its components. We also demonstrated that in such chimaeras the progeny of the 8-cell embryo contribute to all cell lineages of the chimaeric blastocyst; however, the majority of them give rise to TE and PE lineages. Bearing in mind that EPI cells synthesize and secrete FGF4, while FGFR1 and FGFR2 are predominantly expressed in TE and PE cells [14,16,18,22,23,26], we hypothesized that ICM cells can direct the progeny cells of the 8-cell embryo to form the extraembryonic lineages. To assess the involvement of the FGF4/ERK pathway in the plasticity of the chimaeric embryo, we disrupted intercellular interactions between the 8-cell embryo and ICM cells of the E3.5 blastocyst in chimaeras. We employed specific small interfering RNA (siRNA) constructs to perform single (electronic supplementary material, figures S5 and S6; tables S6–S9) and double (figure 5*a*; electronic supplementary material, tables S6, S7 and S10) knockdown of the *Fgfr1* and *Fgfr2* genes in the 8-cell embryo, and as a consequence, blocked the reception of FGF4 secreted by ICM cells.

### 2.6. Preimplantation development of non-chimaeric embryos with double knockdown of *Fgfr1* and *Fgfr2* expression

First, we investigated how the downregulation of *Fgfr1* or *Fgfr2* affects the development of normal, non-chimaeric blastocysts by microinjecting specific siRNA into the zygotes. Having discovered that the downregulation of only *Fgfr1* or only *Fgfr2* had no effect on PE formation (electronic supplementary material, figures S5 and S6; tables S6–S9), we subsequently checked the phenotype of blastocysts with double *Fgfr1* and *Fgfr2* knockdown.

The microinjected embryos were cultured to the 8-, 32- and late E4.5 blastocyst stage and were then subjected to RT-qPCR to assess the maintenance of the silencing after the injection of specific siRNA constructs (figure 5*b,c*; electronic supplementary material, figure S7). We found that in the 8-cell embryos that had developed from siRNA-injected zygotes, *Fgfr1* expression (*n* = 20 in two experiments) decreased by 93% compared to the control siRNA-treated embryos (*n* = 20; *p* = 0.0002; figure 5*b*). In the 32-cell embryos (*n* = 10 in two experiments), the mRNA level for *Fgfr1* was 88% lower than in the embryos injected with non-targeting control siRNA (*p* = 0.030; figure 5*b*). In the E4.5 blastocysts (*n* = 10 in 2 experiments), the expression of the *Fgfr1* was still reduced by 73% compared with controls (*p* = 0.012; figure 5*b*).

We also found a significant decrease in the level of *Fgfr2* mRNA after the injection of specific siRNA. The results showed that in the 8-cell embryos, the introduced siRNA effectively decreased the expression of *Fgfr2* by 85% compared to the embryos microinjected with control siRNA (*p* = 0.0002; figure 5*c*). The efficiency of the *Fgfr2* downregulation gradually decreased with the development of the embryo, and at the 32-cell stage the *Fgfr2* mRNA level was only 43% lower compared to the control siRNA-treated embryos (*p* = 0.045; figure 5*c*). In the E4.5 blastocyst, the relative *Fgfr2* transcript level was only 32% lower compared to the control group (*p* = 0.005; figure 5*c*).

Next, we assessed the developmental competence of *Fgfr1* and *Fgfr2* siRNA-treated embryos (*n* = 25) and compared them with control siRNA-treated embryos (*n* = 22) and non-injected embryos (*n* = 22). The total number of cells in the blastocysts that had developed from zygotes microinjected with siRNA for *Fgfr1* and *Fgfr2* was 64.6 ± 19.7, whereas in the non-injected embryos and the control siRNA-treated embryos, it was significantly higher: 106.7 ± 18.1 cells (*p* < 0.0001) and 105.2 ± 18.4 cells (*p* < 0.0001), respectively (electronic supplementary material, tables S6, S7 and S10). The average proportion of TE cells was similar in all analysed groups (non-injected embryos: 79.2% ± 5.9% cells; control siRNA-treated embryos: 78.4% ± 4.7% cells; *Fgfr1* and *Fgfr2* siRNA-treated embryos: 77.0% ± 9.4% cells; figure 5*d*). Importantly, the simultaneous silencing of *Fgfr1* and *Fgfr2* expression significantly reduced the fraction of PE cells (7.9% ± 3.5%) compared to the corresponding controls (non-injected embryos: 12.3% ± 2.9%; *p* < 0.0001; control siRNA-treated embryos 13.1% ± 3.2%; *p* < 0.0001; figure 5*e*). By contrast, the mean proportion of EPI cells in the *Fgfr1* and *Fgfr2* siRNA embryos was higher (15.1% ± 8.8%) than in the non-injected embryos (8.5% ± 4.2%; *p* = 0.0025) and control siRNA-treated embryos (8.5% ± 3.6%; *p* = 0.002; figure 5*f,g*). We also calculated the results as ICM/TE and PE/EPI ratios (figure 5*h,i*). The SOX17+/SOX2+ inner cells ratio was lower for *Fgfr1* and *Fgfr2* siRNA-treated embryos (0.8) than for non-injected embryos (1.8; *p* = 0.034; Mann–Whitney *U*-test) and control siRNA-treated embryos (1.8; *p* = 0.005). However, there was no change in the ICM/TE ratio (0.3 for all analysed groups).

Based on these data, we conclude that simultaneous downregulation of *Fgfr1* and *Fgfr2* expression impaired the formation of the PE lineage in the blastocyst.

### 2.7. Preimplantation development of aggregates composed of an E3.5 inner cell mass and an 8-cell embryo with double knockdown of *Fgfr1* and *Fgfr2* expression

**Figure 6.**
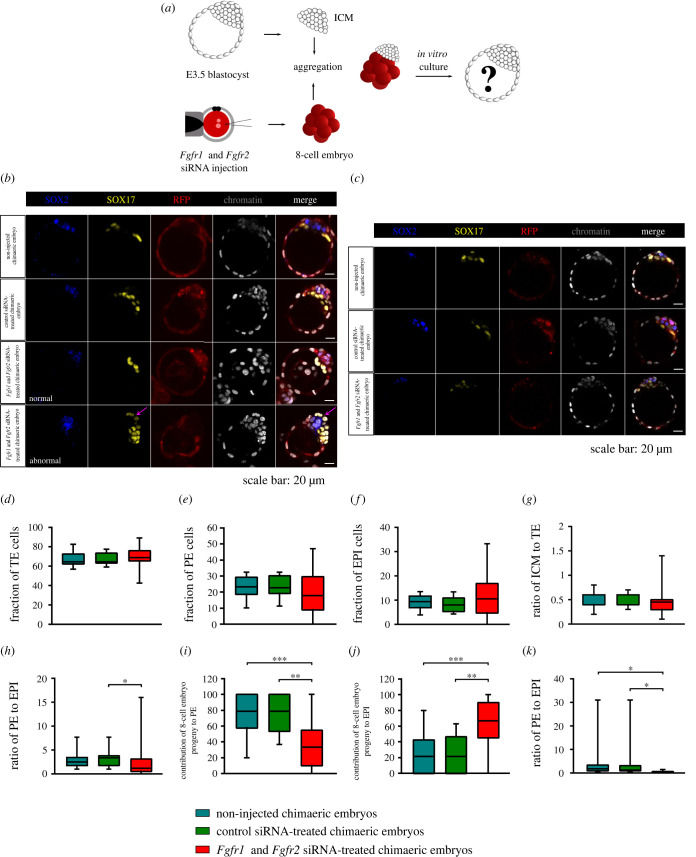
Development of aggregates composed of an E3.5 ICM and 8-cell embryo with double knockdown of *Fgfr1* and *Fgfr2*. (*a*) Experimental outline to assess the effect of inhibiting FGF4/ERK signalling on mouse embryo plasticity. (*b*) SOX2 (blue), SOX17 (yellow) and RFP (red) immunolocalization in representative normal and abnormal chimaeric blastocysts. (*c*) Representative confocal images showing chimaeric blastocysts developed from aggregates composed of two 8-cell embryos in which one of them had reduced *Fgfr1* and *Fgfr2* expression. (*d*–*f*) The percentage contributions of the TE, PE and EPI lineages in chimaeric blastocysts of normal and abnormal morphology. (*g*,*h*) The ICM to TE ratio and the PE to EPI ratio. (*i,j*) The contributions of the microinjected progeny of the 8-cell embryo to the PE and EPI lineages. (K) The PE to EPI ratio. For (*d–k*), the graphs present medians and the first and third quartile values. The ends of the whiskers show the minimum and maximum values. *p < 0.05, **p < 0.01, ***p < 0.001.

With the knowledge that silencing both *Fgfr1* and *Fgfr2* affects PE formation, we constructed chimaeric embryos by aggregating the E3.5 ICM and 8-cell embryo with reduced *Fgfr1* and *Fgfr2* expression (figure 6*a*). The control group contained two types of chimaeric aggregates: the E3.5 ICM and 8-cell embryo that had developed from the zygote injected with non-targeting control siRNA (control siRNA-treated chimaeric embryos) or from the non-injected zygote (non-injected chimaeric embryos).

All chimaeric aggregates of both control groups developed into morphologically normal blastocysts (*n* = 19 for non-injected chimaeric embryos; *n* = 15 for control siRNA-treated chimaeric embryos; figure 6*b*), whereas only half of the *Fgfr1* and *Fgfr2* siRNA-treated chimaeric embryos (22 of 44 embryos) were apparently normal at the late E4.5 blastocyst stage (figure 6*b*). The remaining chimaeric embryos (22 of 44 embryos) developed into abnormal blastocysts, with the PE layer placed outside the embryo, at the site of the TE (figure 6*b*). However, when we created the aggregates composed of two 8-cell embryos, in which one of them had reduced *Fgfr1* and *Fgfr2* expression, they developed into blastocysts with proper morphology (figure 6*c*).

The *Fgfr1* and *Fgfr2* siRNA-treated chimaeric embryos had a lower total number of cells (66.7 ± 21.1) than chimaeric embryos from the corresponding controls (non-injected chimaeric embryos: 122.8 ± 22.0; *p* < 0.0001; control siRNA-treated chimaeric embryos 118.9 ± 18.5; *p* < 0.0001; electronic supplementary material, tables S11–S13). In *Fgfr1* and *Fgfr2* siRNA-treated chimaeric embryos, the fraction of TE cells was on average 68.8% ± 11.4% of all cells, which was similar to the proportion of TE cells observed in the control chimaeric blastocysts (non-injected chimaeric embryos: 67.2% ± 7.2%; control siRNA-treated chimaeric embryos: 67.7% ± 6.3; figure 6*d*). In the case of the average proportions of PE and EPI cells, we also did not notice any differences between the analysed groups (for PE: non-injected chimaeric embryos – 23.7% ± 6.7%; control siRNA-treated chimaeric embryos – 24.0% ± 6.5%; *Fgfr1* and *Fgfr2* siRNA-treated chimaeric embryos – 19.9% ± 14.6%; for EPI: non-injected chimaeric embryos – 9.2% ± 3.2%; control siRNA-treated chimaeric embryos – 8.3% ± 3.3%; *Fgfr1* and *Fgfr2* siRNA-treated chimaeric embryos – 11.3% ± 8.6%; figure 6*e,f*). Additionally, we found that the disruption of intercellular interactions did not affect the ICM/TE ratio (0.5 for all analysed groups; Mann–Whitney *U*-test; figure 6*g*). The PE/EPI ratio was lower for *Fgfr1* and *Fgfr2* siRNA-treated chimaeric embryos (2.7) than for control siRNA-treated chimaeric embryos (3.5; *p* = 0.025; Mann–Whitney *U*-test; figure 6*h*). However, there was no change between the *Fgfr1* and *Fgfr2* siRNA-treated chimaeric embryos (2.7) and non-injected chimaeric embryos (3.0; Mann–Whitney *U*-test; figure 6*h*).

We also checked whether the disruption of intercellular communication between the E3.5 ICM and the 8-cell embryo through FGF receptor knockdown switched the direction of cell differentiation of the 8-cell embryo progeny forming the ICM in the chimaeric aggregate. The results showed that in the *Fgfr1* and *Fgfr2* siRNA-treated chimaeric embryos, the average proportion of 8-cell embryo progeny constituting the PE was 39.3% ± 36.3%, and it was much lower than in the chimaeric aggregates from both control groups (non-injected chimaeric embryos: 76.1% ± 25.2%; *p* = 0.0006; control siRNA-treated chimaeric embryos: 73.9% ± 24.1%; *p* = 0.003; Mann–Whitney *U*-test; figure 6*i*). Conversely, 8-cell embryo progeny more readily contributed to SOX2+ EPI than in the corresponding control chimaeric blastocysts (non-injected chimaeric embryos: 23.9% ± 25.2%; *p* = 0.0006; control siRNA-treated chimaeric embryos: 26.1% ± 24.1%; *p* = 0.003; *Fgfr1* and *Fgfr2* siRNA-treated chimaeric embryos: 60.7% ± 36.3%; Mann–Whitney *U*-test; figure 6*j*). There was also a reduced SOX17+/SOX2+ inner cells ratio for *Fgfr1* and *Fgfr2* siRNA-treated chimaeric embryos (0.4) compared to the corresponding controls (non-injected chimaeric embryos – 4.5; *p* = 0.017; control siRNA-treated chimaeric embryos – 4.6; *p* = 0.013; Mann–Whitney *U*-test; figure 6*k*).

Taken together, our results demonstrate that impaired FGF-dependent intercellular signalling leads to a switch between PE and EPI fates, thus resulting in a reversal of the proportion of 8-cell embryo-derived cells in the ICM and abnormal development of the chimaeric embryo. Hence, the FGF4/ERK pathway, through FGFR1 and FGFR2 receptors acts as a cell fate regulator promoting the proper formation of all cell lineages, necessary for the successful development of the embryo in the uterus.

## 3. Discussion

The gradual restriction of ICM cells potency and the reduced plasticity of the whole embryo have been widely studied, but the results of experiments have differed depending on the applied experimental approach [8]. Using as a model chimaeras constructed by aggregating the ICM of the E3.5 or E4.5 blastocyst with an 8-cell embryo, which retains the potential to differentiate into all primary cell lineages, allowed us to examine the plasticity of PE and EPI precursor cells within the ICM niche and the mechanisms behind the self-organization of the embryo.

First, we confirmed that ICMs derived from both early and late blastocysts no longer have the capacity to differentiate into the TE lineage and preferentially form the EPI and PE in the chimaeric embryo. This result is in accordance with the generally accepted view of the potency of blastocyst ICM cells [1–4,7,19,29,30]. Importantly, all of these authors' observations, resulting from various experimental approaches, support the notion that the plasticity of ICM cells depends not only on the developmental stage of the blastocyst from which they are derived but also on the microenvironment (niche), defined as the interactions between neighbouring cells or another source of positional information. The significance of the interactions of ICM cells with extracellular matrix *via* integrins in their regulatory behaviour has been recently directly demonstrated by Kim *et al*. [2], who compared the development of isolated ICMs cultured in KSOM medium with the ICMs, which were provided with extracellular matrix by embedding in Matrigel [2].

Our experiments regarding chimaeras composed of an E3.5 ICM and 8-cell embryo also revealed that despite the lost ability of the ICM to differentiate into the TE, aggregates composed of these two components formed properly built blastocysts comprising three cell lineages, thus demonstrating their ability to self-organize. Their correct development was assured by the presence of the accompanying partner, which was able to rescue TE development. Our results have been confirmed by the postimplantation development of these embryos, which ended with the birth of the young. As predicted, the bodies of the resulting fetuses and yolk sac were built of both components, whereas the placenta—a TE derivative—originated exclusively from the 8-cell embryo progeny. However, our results contradict those of Rossant & Lis [31], who showed that ICMs isolated from 32- to 50-cell blastocysts and aggregated with 8-cell mouse embryos give rise to TE-derived tissues during postimplantation development. Moreover, the aggregation of two ICMs at a similar stage and the subsequent transplantation of such aggregates into the recipient female resulted in the formation of a normal E5.5 egg cylinder [31]. However, it is worth noting that the ICMs used in their experiments originated from less advanced blastocysts than those used in our study. It also cannot be ruled out that the observed reconstruction of the TE layer was a result of an inefficient ICM isolation procedure, which allowed some TE cells to survive on the ICM surface [6].

We also observed that in contrast with the chimaeras with an E3.5 ICM, chimaeras consisting of an E4.5 ICM (about 110 cells) aggregated with an 8-cell embryo had limited developmental potential. The resulting chimaeric embryos were able to successfully undergo whole embryogenesis, culminating in the birth of the young, provided that the blastomeres of the 8-cell embryo were in direct contact with the PE layer. When they adjoined only the EPI layer of the ICM, the development of these aggregates failed, and, as a consequence, the transfer of such chimaeric aggregates into the reproductive tracts of recipient females resulted only in resorptions. The developmental potential of embryos constructed by aggregating E4.5 ICMs and 8-cell embryos was also examined by Rossant [32]. However, this author combined both components randomly and did not analyse their development to term. In egg cylinders and E9.5 and E10.5 fetuses, ICM cells of late blastocysts gave rise only to embryonic tissues, which suggests that they were not involved in TE formation [32]. Rossant also noted a decreased implantation rate of the chimaeric aggregates with an E4.5 ICM compared to the control chimaeric aggregates involving an E3.5 ICM [32]. However, since she did not analyse the preimplantation development of the chimaeric embryos, she was not able to find the cause(s) of this phenomenon. Our research shows that chimaeras composed of 8-cell embryos aligned with the EPI failed to develop, in contrast with those in which the 8-cell embryos had contact with the PE. All observed abnormalities concerned the incorrect localization of the PE layer, which maintained its original outer position, thus partially occupying the place of the TE layer in the chimaeric blastocyst. Since the TE gives rise to the embryonic part of the placenta and is responsible for the contact between the blastocyst and the uterine endometrium, it seems likely that disturbances in its development may be the reason for the low rate of implantation and birth observed in our experiments and in Rossant's study [32]. Cells of both extraembryonic cell lineages, TE and PE, undergo polarization and acquire an epithelial-like character. Proteins such as LRP2 (*lipoprotein receptor-related proteins 2*) and DAB2 (*disabled-2*) accumulate in the apical part of PE cells [33], whereas proteins of tight junctions (including claudins, occludins and cingulin) and polarization proteins (PARD3/PARD6B/aPKC) are present on the lateral-apical surface of TE cells [34]. It seems therefore unlikely that a layer of polarized TE cells would be able to cover the PE layer, which is also polarized. To verify whether the loss of chimaeric embryo plasticity is related to the presence of a polarized PE layer originally directed outwards, we aggregated an 8-cell embryo with the ICM of an E4.5 blastocyst devoid of PE cells. ICMs containing exclusively EPI cells were obtained from the blastocysts that had developed from the 8-cell stage in medium supplemented with FGF4/ERK signalling pathway inhibitors targeting FGFR and MEK kinase [20,21]. The EPI of such embryos corresponds to the mature, functional EPI of E4.5 control blastocysts, as evidenced by gene profile, epigenetic status and functional studies [20]. The successful development of such chimaeras until the late blastocyst stage led us to conclude that ICM plasticity decreases as PE cells segregate and polarize on the ICM surface. However, the developmental potential of chimaeras made of this component is unimpaired only if the PE layer is surrounded by apolar cells with an unrestricted ability to differentiate.

A signalling pathway potentially involved in regulating chimaeric mouse embryo development is the FGF4/ERK cascade. Its role in the formation of two ICM cell lineages, EPI and PE, has been thoroughly demonstrated [10–12,15,20,21,35]. ICM cells differ in the level of FGF4 expression in such a way that EPI precursor cells are characterized by a higher expression of this gene than PE precursor cells [13,14,16]. Additionally, it has been shown that the FGFR1 and FGFR2 receptors are located on both PE and TE cells [18,22,36]. Such specific expression of ligand and receptors in different lineages of the embryo indicates that paracrine interactions involving this pathway may underlie the plasticity of the mouse embryo. To address this question, we experimentally disrupted the communication between both components of the chimaera by interfering with the transduction of this pathway: we performed a double knockdown of *Fgfr1* and *Fgfr2* genes in an 8-cell embryo [16,18].

We revealed that in the absence of the ability to respond to the ICM cells' FGF4 signal, 8-cell embryo progeny preferentially contributed to the EPI rather than PE lineage, in contrast with chimaeric embryos with an undisturbed signalling pathway, where the ICM-derived FGF signal triggered the differentiation of neighbouring 8-cell embryo progeny mostly towards the PE lineage, thereby ensuring a balanced cell-type composition. Importantly, regardless of the origin of the cells contributing to the PE and EPI, the proportions of these two lineages were maintained and corresponded to those found in the control chimaeric embryo. This balance in EPI and PE proportions is known to be critical for the development of the embryo beyond implantation. Using *in silico* simulations supported by experimental manipulation of embryo size, Saiz *et al*. [37] revealed that in response to an increase or decrease of the cell number of one lineage (PE or EPI), the differentiation pattern of uncommitted progenitor cells is changed to restore the lineage composition balance [37]. To show the involvement of FGF in this regulatory mechanism, they combined *Fgf4*^−/−^ embryos with embryonic stem cells and concluded that this growth factor is responsible for coupling lineage size with the cell fate decisions of uncommitted progenitor cells [37]. This result agrees with our results, which confirm that paracrine interactions mediated by the FGF4/ERK pathway are necessary to maintain the correct proportions of PE and EPI cells within the ICM of the developing blastocyst.

Moreover, we also noted that a decrease of FGFR1/2 expression in 8-cell embryo-derived cells resulted in incorrect cell lineage formation in half of the chimaeric blastocysts. These blastocysts showed abnormalities of the TE, which were manifested by the mislocalization of the PE cell layer in the place of the TE. Since FGFR1 and FGFR2 receptors have also been identified in the TE [14,22,38], it seems that the FGF4 secreted by ICM cells can also regulate TE development in a paracrine manner. It has been previously shown that only uncommitted target cells may be sensitive to the FGF signal, reacting appropriately by changing their fate [4]. Reduced levels of FGF receptors prevented the transmission of the signal to the target 8-cell embryo blastomeres and the self-organization of the developing chimaeric embryo. The phenotype of our chimaeric embryos suggests that the abnormalities were probably due to impaired proliferation rather than a specification of the TE lineage. We suppose that as a result of reduced proliferation, the TE cells were unable to spread over the entire surface of the developing blastocyst and therefore could not prevent PE cells from polarization, thus disturbing the integrity of the TE layer.

In contrast with PE formation, there are discrepancies regarding the effect of FGF4/ERK signalling on the TE lineage. It was shown that *Fgf4*^−/−^ mutants have decreased FGFR2 expression in the TE of E4.5 blastocysts [39]. On the other hand, treating zygotes with inhibitors of the FGF pathway does not impede cavitation and formation of the blastocyst containing a CDX2-expressing outer epithelial layer [20]. Another study showed that culturing embryos at the morula stage in a medium supplemented with FGF/ERK inhibitors does not affect TE development, but blocking this pathway at an earlier stage, in the 8-cell embryo, results in a disturbance of CDX2 protein expression, inhibition of the cavitation process and developmental delay [40]. This discrepancy was due to the differences in ERK2 distribution between these stages, highlighting its importance in maintaining the TE identity [40]. Another study, using *Fgfr1*^−/−^ mutants, also points to the role of the FGFR1 receptor in TE development [23]. These knockout embryos showed abnormalities in cell polarity, which were manifested, among other ways, by the incorrect localization of E-cadherin [23]. Prior to the implantation of *Fgfr1*^−/−^ embryos, cells of the mural TE do not downregulate *Cdx2* expression, which is necessary to initiate their differentiation into trophoblast giant cells and the implantation of the embryo in the uterine endometrium [23]. By contrast, there are reports showing that inhibiting the FGF4/ERK pathway results in a reduced number of TE cells compared to control embryos [20]. The same phenotype was observed in the case of *Fgfr1^−/−^; Fgfr2^−/−^* double mutants [18]. While the specification of the TE is not generally impaired in these mutants, the proportion of cells contributing to this lineage is decreased compared to the wild-type embryos [16,18], suggesting the involvement of this signalling pathway in the expansion rather than the specification of the TE. The results of the above studies suggest that the abnormal morphology of half of the aggregates of the E3.5 blastocyst and the 8-cell embryo with reduced expression of the *Fgfr1* and *Fgfr2* genes may result from disturbances in the proliferation of TE cells due to the disruption of cell communication mediated by these receptors. Since the second component (the ICM) is unable to compensate for the deficiency of this lineage due to the loss of totipotency, as we showed in our study, the development of the entire chimaeric embryo fails. To resolve this issue, we decided to aggregate an 8-cell embryo subjected to double knockdown of *Fgfr1* and *Fgfr2* with the intact component (i.e. the control 8-cell embryo, which retains its ability to form the TE). The development of such embryos turned out to be successful and resulted in the formation of normal blastocysts comprising a TE made up of the daughter cells of both aggregate components. This finding suggests that cell communication mediated by FGFR1 and FGFR2 receptors may be necessary for the proper expansion of the TE in the blastocyst. However, our experiment does not resolve whether this signalling is paracrine or autocrine in nature. There is a report showing that the FGF2 factor secreted by TE cells binds to the FGFR2 receptor and *via* the PKC/p38 MAPK (*protein kinase C/mitogen-activated protein kinase*) pathway affects, in an autocrine manner, the expression and localization of E-cadherin, ZO-1 (*zonula occludens-1*), and the sodium-potassium pump of ATPase or aquaporins 3 and 9, which are related to the cavitation process [36]. These authors injected *Fgfr2* siRNA into the cavity gap between the *zona pellucida* and the TE of the blastocyst and found that, as a consequence of knockdown, blastocyst expansion was significantly compromised [36]. This effect seems to be more severe than that in our experiment. However, it is worth noting that Yang *et al*. [36] induced knockdown later in development (at the blastocyst stage) and used a transfection method rather than the microinjection of zygote method applied in our study, which could contribute to these discrepancies. Interestingly, p38 MAPK signalling involved in the regulation of expanded blastocyst formation has also been shown to be important for early PE specification from uncommitted ICM progenitors [41–44], which further speaks to the similarity between the specifications of both extraembryonic lineages: the TE and PE.

It remains unclear why the abnormalities related to the structure of the blastocyst constructed with an E3.5 ICM and 8-cell embryo subjected to FGFR1/2 knockdown affected only half of the embryos analysed. One possible reason may be that the efficiency of silencing *Fgfr2* gene expression decreased as the embryo developed. We found that although *Fgfr2* mRNA was almost fully degraded in experimental embryos at the 8-cell stage, already at the 32-cell stage, *Fgfr2* expression was reduced only to half of the level found in the control embryos, and at the late E4.5 blastocyst stage, the relative amount of *Fgfr2* transcripts was only one-third lower than that of the control group at this stage. By contrast, the silencing efficiency of *Fgfr1* was high up to the blastocyst stage. Therefore, it seems possible that the differences in morphology of the chimaeric blastocysts were due to the varied efficiency of FGFR1/2 knockdown.

**Figure 7.**
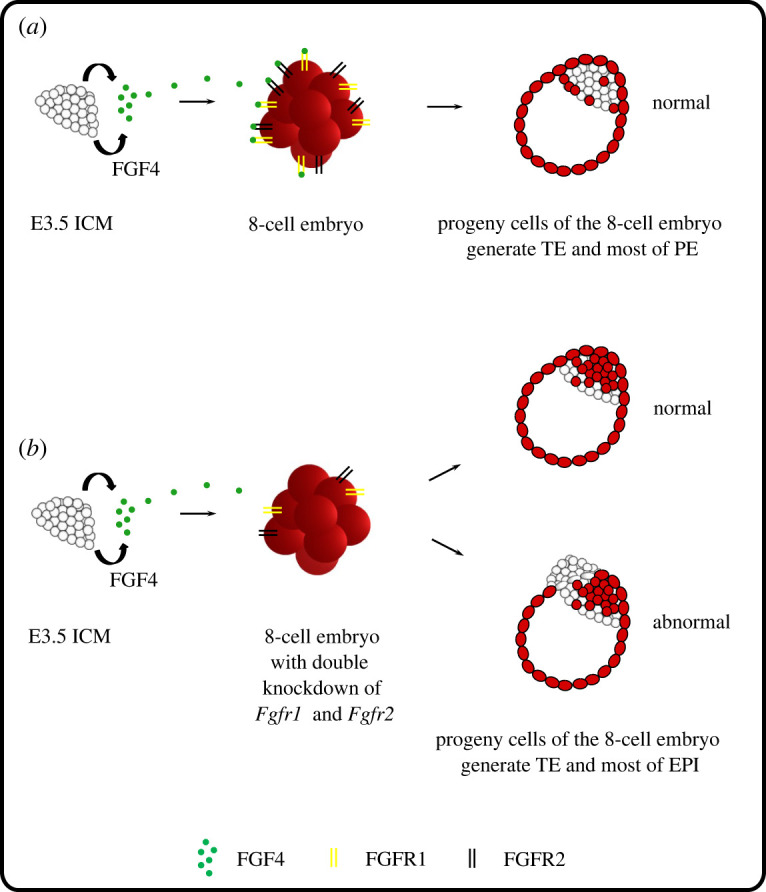
Working model of the role of FGF4/ERK signalling on mouse embryo development and its plasticity. (*a*) During the development of aggregates composed of an E3.5 ICM and 8-cell embryo, the FGF4/ERK cascade, acting *via* FGFR1 and FGFR2, drives the progeny of the 8-cell embryo to adopt a TE and PE identity. (*b*) The disruption of intercellular communication between the E3.5 ICM and the 8-cell embryo through FGF receptor knockdown results in a preferential contribution of the 8-cell embryo progeny to the TE and EPI lineages and impaired development of the chimaeric blastocyst.

Taken together, our results clearly show that despite the limited developmental potential of individual components that make up the aggregate, the blastomeres of the resulting chimaeric embryo communicate with each other *via* FGF signalling to guarantee the precise proportions of cell lineages required for successful embryogenesis until term. Disruption of intercellular signalling mediated by FGFR1 and FGFR2 receptors results in inversion of the distribution of 8-cell embryo progeny to the PE and EPI and possibly impairs the development of the TE (figure 7). Thus, the FGF signalling pathway is required for coordinating cell fate decisions to ensure an appropriate ICM composition and TE integrity. However, the activity of this signalling must be strictly regulated temporally and spatially for the proper formation of the cell lineages in the blastocyst. It is worth noting that the successful integration of both components into one chimaeric embryo despite the disruption of FGF signalling indicates that additional mechanisms, such as geometrical confinement and/or intrinsic feedback interactions between mechanical and biochemical factors, may play the role in its self-organization [9]. It also suggests that the strategy, applied by the embryo to regulate its development and the complexity of the involving regulatory mechanism, may depend on the type of experimental perturbation (i.e. on the nature of the components that make up the chimaera).

## 4. Materials and methods

### 4.1. Animals

Mice were kept at a 14 h light:10 h dark regime. F1(C57BL/6/Tar × CBA/Tar) females and Tg(CAG-DsRed*MST)1Nagy/J [45,46], Pdgfr*α*H2B-EGFP [47], C57BL/6-Tg(UBC-GFP)30Scha/J or F1(C57BL/6/Tar × CBA/Tar) males were used in this study. F1(C57BL/6/Tar × CBA/Tar) female mice, which were used as recipients of the chimaeric embryos, were mated with the vasectomized F1(C57BL/6/Tar × CBA/Tar) males.

### 4.2. Recovery of zygotes

To obtain zygotes, F1(C57BL/6/Tar × CBA/Tar) females were induced to superovulation with 10 IU of PMSG (pregnant mare's serum gonadotropin; Intervet) followed after 48 h with 10 IU of hCG (human chorionic gonadotropin; Intervet) and were mated with F1 or Tg(CAG-DsRed*MST)1Nagy/J males. Females, in which vaginal plugs were detected, were autopsied 22 h after hCG injection. Zygotes were recovered from the ampullae of oviducts and cleared of follicular cells using hyaluronidase (300 µg ml^−1^, Sigma-Aldrich).

### 4.3. Recovery of 8-cell embryos

Eight-cell embryos were obtained from spontaneously ovulating F1(C57BL/6/Tar × CBA/Tar) females crossed with Tg(CAG-DsRed*MST)1Nagy/J) or F1(C57BL/6/Tar × CBA/Tar) males. Females were screened for vaginal plugs the following morning (E0.5). Eight-cell embryos were flushed from the oviducts and uteri of females on the 3rd day post coitum (dpc) (about 58 h after mating) with a standard medium supplemented with 4 mg ml^−1^ BSA (bovine serum albumin; Sigma-Aldrich) and collected in small droplets of this medium under mineral oil at 37.5°C in 5% CO_2_ in the air.

### 4.4. Recovery of blastocysts

To obtain E3.5 (64-cell stage) and E4.5 (100-cell stage) blastocysts, spontaneously ovulating F1(C57BL/6/Tar × CBA/Tar) females were crossed with PDGFR*α*H2B:EGFP or C57BL/6-Tg(UBC-GFP)30Scha/J males. Females with vaginal plugs were autopsied on the 4th dpc, and E3.5 blastocysts were collected by flushing oviducts and uteri with M2 medium supplemented with BSA. Late (E4.5) blastocysts were obtained as a result of 24 h of *in vitro* culture of E3.5 blastocysts.

### 4.5. Treatment of embryos with FGF4/ERK inhibitors

To obtain blastocysts devoid of the PE lineage, 8-cell embryos were treated with a combination of a MEK kinase inhibitor (PD0325901, 500 nM, Stemgent) [20,21] and an FGF receptor inhibitor (PD173074, 100 nM, Stemgent) in KSOM medium [21] for 72 h.

### 4.6. Inner cell mass isolation (immunosurgery)

The *zonae pellucidae* were removed from E3.5 or E4.5 blastocysts using acidic Tyrode's solution [48] (10–30 s). The blastocysts were then placed in anti-mouse serum (1 : 3, 30 min, Sigma-Aldrich) followed by incubation in the guinea pig complement (1 : 3, 30 min, Sigma-Aldrich) [49]. Lysed TE cells were removed by pipetting.

### 4.7. Formation of chimaeras

*Zona pellucida*-free 8-cell embryos and ICMs of E3.5 and E4.5 blastocysts were aggregated in M2 medium supplemented with phytohemagglutinin (PHA, 300 µg ml^−1^, Sigma-Aldrich), rinsed in M2 and cultured in KSOM medium [50] (Merck Millipore) at 37.5°C, 5% CO_2_ in the air under mineral oil for 24 or 48 h. Aggregation of the 8-cell embryo with the ICM of a late E4.5 blastocyst was performed under a fluorescent stereoscopic microscope (Olympus SZX16). In such aggregates, the 8-cell embryos were attached in a controlled manner in a position adjacent to the PE or EPI layer of the ICM expressing GFP protein under the control of the *Pdgrfα* gene promoter, which is active only in PE cells. Both types of aggregates were then cultured as described above.

### 4.8. Time-lapse imaging

Time-lapse imaging of chimaeric aggregates was performed using an inverted fluorescence microscope (Axio Observer Z1, Carl Zeiss) equipped with an incubation chamber ensuring a temperature of 37.5°C and 5% CO_2_ in air (Incubator XL multi S1, Carl Zeiss) and a camera (AxioCam HR R3, Carl Zeiss). Single embryos were placed in drops of KSOM medium under mineral oil on a glass-bottomed dish, and their development was recorded using a 40x objective (EC Plan-Neofluar 40x/0,75 M27) for 48 h. For eGFP fluorescence, the wavelength commands used were 488 nm excitation and 509 nm emission. For DsRed, the settings were adjusted to a 590 nm excitation wavelength and a 612 nm emission wavelength. Three-channel (eGFP, DsRed and transmitted light) images were acquired every 15 or 20 min, every 2.5 µm. ZEN 2012 software (blue edition, Carl Zeiss) was used for analysis.

### 4.9. Immunostaining

Chimaeric blastocysts were fixed in 4% paraformaldehyde (30 min, room temperature [RT], ThermoFisher Scientific), permeabilized in phosphate-buffered saline (PBS) containing 0.5% Triton X-100 (30 min, RT, Sigma-Aldrich) and blocked in PBS with 10% fetal bovine serum (FBS, ThermoFisher Scientific) overnight at 4°C. In the case of aggregates composed of both GFP- and RFP-expressing cells, only the TE or PE marker was labelled using mouse monoclonal antibody against CDX2 (1 : 50, BioGenex, MU392A-UC) or goat polyclonal antibody against GATA4 (1 : 100, R&D Systems, AF2606), respectively. In the case of aggregates consisting of only RFP-expressing cells, markers of both lineages were detected. In aggregates composed of only GFP-positive and GFP-negative components markers of SOX17 and SOX2 were used (rabbit polyclonal antibody against SOX2; 1 : 100, R&D Systems, AF1924 and goat polyclonal antibody against SOX17; 1 : 100, Abcam, ab97959). After 24 h of incubation at 4°C, the embryos were rinsed with PBS and incubated with a mixture of corresponding secondary antibodies: FITC-conjugated goat anti-mouse IgG (1 : 200, Jackson ImmunoResearch Laboratories, Inc., 115-095-146) or Alexa 633-conjugated rabbit anti-mouse IgG (1 : 200, ThermoFisher Scientific, A21063), Alexa Fluor 488 donkey anti-goat IgG (1 : 200, ThermoFisher Scientific, A11055) and Alexa 633-conjugated goat anti-rabbit IgG (1 : 200, ThermoFisher Scientific, A21071) for 2 h at RT and rinsed again. Antibodies were diluted in a blocking solution. To visualize the nuclei, the blastocysts were stained in microdroplets of chromomycin A_3_ (0.01 mg ml^−1^ with 5 mM MgCl_2_ in PBS, Sigma-Aldrich) on glass-bottomed dishes (MatTek Corporation) for 30 min at 37.5°C. In the case of blastocysts with knockdown of FGFR1 and/or FGFR2, the FGFR2 and markers of all three cell lineages (CDX2 for TE, SOX2 for EPI, and SOX17 or GATA4 for PE) were immunostained. The following primary and secondary antibodies were used: rabbit polyclonal antibody against FGFR2 (1 : 200, Santa Cruz Biotechnology, Bek antibody (C-17): sc-122), rabbit polyclonal antibody against SOX2 (1 : 100, Abcam, ab97959), goat polyclonal antibody against SOX17 (1 : 100, R&D Systems, AF1924), Alexa Fluor 647 donkey anti-rabbit IgG (1 : 200, ThermoFisher Scientific, A31573) and Alexa Fluor 488 donkey anti-goat IgG (1 : 200, ThermoFisher Scientific, A11055).

### 4.10. Confocal analysis

Confocal images were acquired with an LSM 510 Zeiss inverted confocal microscope (Jena, Germany). Z-stacks of 60 optical sections for blastocysts were collected. The number of nuclei in embryos was counted, and the correlation between the presence or absence of GFP/RFP marker and lineage-specific markers was recorded. Images were analysed with LSM Image Browser, and ImageJ software was used to count cells.

### 4.11. Transfer of chimaeric embryos

After 24 h of *in vitro* culture, the chimaeric blastocysts were transferred by standard methods to the oviducts of F1 recipient females obtained by mating with vasectomized F1 males of proven sterility [51,52]. We transferred approximately 10 embryos into the right oviduct of the recipient on the first day of pseudopregnancy. Pregnancies were controlled by checking vaginal smears starting on the 11th day after transfer. If the smear showed signs of approaching oestrus, the female was autopsied to recover possible remnants of the resorbed embryo. Implantation sites, identified as clearly visible marks on the uterus devoid of fetuses, were referred to as resorptions.

### 4.12. Dissection of newborns and their organs

Recipient females were autopsied on the 19th day of pregnancy or left until parturition, and the number of fetuses or newborns was counted. The placenta and fetal membranes were separated from the embryo. To evaluate the extent of the chimaerism quantitatively (by the presence of GFP and RFP fluorescence), the organs of the offspring (lungs, heart, skin, intestine, brain, kidneys, liver and spleen) were isolated, cut into pieces and dissociated into a suspension of cells in TrypLE Express using a gentleMACS Dissociator (Miltenyi Biotec) according to the manufacturer's protocol. Individual cells were examined by fluorescence microscope (Zeiss Axiovert 135). Images taken in five places of the preparation were analysed for the number of GFP-expressing and RFP-expressing cells. The yolk sac was placed in a mixture of enzymes: 0.5% trypsin (BDH Chemicals, Merck) and 2.5% pancreatin (Sigma-Aldrich) for 3 h at 4°C. They were then stored overnight in M2 medium at 4°C. On the next day, two layers of the yolk sac—the mesoderm and endoderm—were separated and disaggregated by pipetting after 15 min of incubation at 37.5°C in small droplets of TrypLE Express (ThermoFisher Scientific). The placenta was assessed only macroscopically.

### 4.13. Knockdown of FGFR1 and FGFR2 expression

To downregulate *Fgfr1* and *Fgfr2*, we used siRNA against mRNA for FGFR1 (Silencer Select Pre-designed siRNA, s66023, Ambion) and FGFR2 (Silencer Select Pre-designed siRNA, s201347, Ambion). The specific siRNA were co-injected at a concentration of 10 µM with the fluorescent marker BLOCK-iT Alexa Fluor Red Fluorescent (1 µM in RNA-free water; ThermoFisher Scientific) into the mouse zygotes. As a fluorescent marker we used non-specific, double-stranded RNA (dsRNA) conjugated with Alexa 555. The knockdown of *Fgfr1* or *Fgfr2*, and double knockdown were performed. As controls, embryos injected with scrambled siRNA (10 µM; Silencer Select Negative Control No. 2 siRNA, Ambion) and embryos not subjected to microinjection were used. Microinjection was performed using a FemtoJet or CellTram injector (Eppendorf) under an inverted Zeiss Axiovert 200 microscope equipped with TransferMan Eppendorf micromanipulators. Two types of pipettes were used (Borosilicate Glass Capillaries, Harvard Apparatus): holding pipettes (1.0 mm and 0.5 mm—outer and inner diameter, respectively) and injection pipettes (1.0 mm and 0.78 mm—outer and inner diameter, respectively). Microinjections were carried out in micro-drops of M2 medium under mineral oil. Next, the embryos were transferred to droplets of M2 medium under mineral oil on the glass-bottomed dish to determine the microinjection efficiency under the fluorescent microscope. They were subsequently cultured for 48, 72 or 96 h in KSOM medium in standard conditions.

The embryos at the 8-cell stage with knockdown of *Fgfr1* and *Fgfr2* were aggregated with the ICM of the early blastocyst and cultured in KSOM medium for 48 h in standard conditions.

### 4.14. RT-qPCR

mRNA was isolated using the Dynabeads mRNA DIRECT Micro kit (ThermoFisher Scientific) according to the manufacturer's protocol. Samples (10 embryos at the 8-cell stage, 5 embryos at the 32-cell and the blastocyst stage; repeated twice) were lysed in 20 µl of lysis/binding buffer and frozen at −80°C. To capture mRNA, 10 µl of paramagnetic oligo-(dT)25 bead suspension was used, and the mixture was rotated for 30 min at RT (Thermo-Shaker TS-100, Biosan). mRNA was eluted from the beads by adding 10 µl of DEPC-treated water and heated for 10 min at 70°C with 0.5 µg Oligo(dT)12–18 (ThermoFisher Scientific). The reverse transcription was performed in a total volume of 20 µl using 1xRT buffer, 10 mM DTT, 200 U of Superscript II Reverse Transcriptase, 0.5 mM dNTPs and 40 UI RNase inhibitor (ThermoFisher Scientific). Reverse transcription was performed at 42°C for 50 min and subsequently at 70°C for 15 min. Synthesized cDNA was diluted two times with DNAse and RNAse-free water (ThermoFisher Scientific). TaqMan Gene Expression assays in the OneStep Real-Time PCR System were used. The list of TaqMan assays, specific to the studied genes, is presented in the electronic supplementary material, table S14. Reactions were set in 10 µl volume, and the amplification was run for 50 cycles: 95°C for 15 s, 60°C for 1 min. The relative level of expression was evaluated using the 2^−ΔCt^ method [53], where actin B (ActB) was used for normalization [54].

### 4.15. Statistical analysis

Statistical analysis was conducted using IBM SPSS Statistics 23. The basic descriptive statistics were analysed together with the Shapiro–Wilk test and the Student's *t*-test for independent samples. The Mann–Whitney *U*-test was used to compare the ICM/TE and PE/EPI ratios. *p*-values of less than 0.05 were considered statistically significant.

## Data Availability

The data are provided in the electronic supplementary material [55].
